# Meta-Analytically Informed Network Analysis of Resting State fMRI Reveals Hyperconnectivity in an Introspective Socio-Affective Network in Depression

**DOI:** 10.1371/journal.pone.0094973

**Published:** 2014-04-23

**Authors:** Leonhard Schilbach, Veronika I. Müller, Felix Hoffstaedter, Mareike Clos, Roberto Goya-Maldonado, Oliver Gruber, Simon B. Eickhoff

**Affiliations:** 1 Department of Psychiatry, University Hospital Cologne, Cologne, Germany; 2 Institute of Neuroscience and Medicine (INM-1), Research Centre Juelich, Juelich, Germany; 3 Cognitive Neuroscience Group, Institute of Clinical Neuroscience and Medical Psychology, University of Duesseldorf, Duesseldorf, Germany; 4 Center for Translational Research in Systems Neuroscience and Psychiatry, Department of Psychiatry and Psychotherapy, Georg August University Goettingen, Goettingen, Germany; Universiteit Gent, Belgium

## Abstract

Alterations of social cognition and dysfunctional interpersonal expectations are thought to play an important role in the etiology of depression and have, thus, become a key target of psychotherapeutic interventions. The underlying neurobiology, however, remains elusive. Based upon the idea of a close link between affective and introspective processes relevant for social interactions and alterations thereof in states of depression, we used a meta-analytically informed network analysis to investigate resting-state functional connectivity in an introspective socio-affective (ISA) network in individuals with and without depression. Results of our analysis demonstrate significant differences between the groups with depressed individuals showing hyperconnectivity of the ISA network. These findings demonstrate that neurofunctional alterations exist in individuals with depression in a neural network relevant for introspection and socio-affective processing, which may contribute to the interpersonal difficulties that are linked to depressive symptomatology.

## Introduction

Depression is a highly prevalent mental disorder, whose underlying neurobiology is still only partially understood. Affective symptoms such as depressed mood, loss of interest and enjoyment, and reduced energy leading to increased fatigability and diminished activity constitute the psychopathological core of the disorder. Furthermore, depression is characterized by abnormally increased introspective thoughts and self-referential concerns [1], [2], which may contribute to dysfunctional interpersonal expectations and make successful participation in social interaction difficult [3], [4]. Since social interactions are normally experienced as intrinsically rewarding [5], unsuccessful or reduced social interactions can further contribute to depressive symptomatology and eventually its chronification [4], [6]. The neurobiology that may mediate the relationship between affective, self-referential and introspective processes, however, is yet poorly understood.

Several recent findings point towards aberrant functional connectivity between specific brain regions in depression [7], [8] and have provided evidence for an alteration of cortico-limbic connections in individuals vulnerable to this disorder [9]. Resting state fMRI analyses of functional connectivity provide a powerful approach to investigate network dysfunctions in depression. In particular and contrast to task-based neuroimaging, such analyses are less confounded by cognitive and/or motivational impairments, which are commonly observed in patient populations and impair sufficient task performance [7]. Yet, the reliable identification of functional connectivity networks is complicated by various methodological drawbacks [10]: While data-driven approaches allow a robust definition of networks of covariant activity, their definition is directly dependent on the used data. In seed-based approaches, on the other hand, the observed patterns of functional connectivity often depend upon the choice of the seed regions and, hence, potentially open to bias (e.g. by using regions from a single previous study or placing the seeds by hand). Moreover, in both of these approaches functional meaning is usually assigned to the derived networks by reverse inference, i.e. inferring the presence of certain cognitive processes based on the involvement of certain brain regions [11]. Neither approach, therefore, allows investigating aberrant functional connectivity in a brain network, whose derivation is based on *a priori* hypotheses and has been carried out in an unbiased manner.

The present study aims to circumvent these methodological problems by applying a hypothesis-driven, model-based approach to the analysis of functional connectivity alterations in depression: In particular, we used inter-regional correlations in resting state fMRI data to estimate connectivity within a meta-analytically derived network model. Such an approach has the major advantage of targeting a neural network that was defined by statistical convergence across findings of hundreds of previous neuroimaging studies. Given the above described considerations on the pathophysiology of depression and its relationship to self-referential and affective processes, we focused on those parts of the so-called “default mode network” that are also engaged by affective processing. While the default mode network plays a major role in self-referential thoughts and introspection [12], the “affective network” is especially relevant for emotion perception and regulation [13]. As both aspects not only contribute to social cognition, but are also likely to be disturbed in depression, we examined the combination of both circuits, the conjunction of which we refer to as the “introspective socio-affective” (ISA) network. The ISA network defined by a recent large-scale meta-analysis of functional neuroimaging studies comprises anterior and subgenual cingulate regions relevant for interpersonal action control and the generation of predictions concerned with another person's behavior [14]. Furthermore, it includes the dorsomedial prefrontal cortex and the precuneus, both of which have been implicated in mental state attribution, autobiographic memory as well as prospective meta-cognition [12], [13]. Lastly, the ISA network includes the amygdala, whose role in fear conditioning, anxiety and relevance-detection has been well established (for a review see [15]).

Taken together, the current study is based on the idea that aberrant functional connectivity in a neural network for affective, introspective and social processing may represent a key pathophysiological aspect of depressive symptomatology. To investigate this, resting state connectivity in a group of patients with depression and a cohort of matched controls was examined in a meta-analytically derived, robust *a priori* network model. In line with the “hyperconnectivity hypothesis” of depression [16], we hypothesized to find more pronounced neurofunctional coupling within the ISA network in depressed patients as compared to healthy controls.

## Methods

### Ethics statement

All subjects gave written consent to participate in the study as approved by the ethics committees of the University of Aachen and the University of Goettingen. The ethics committee at the HHU Düsseldorf approved joint re-analysis.

### Meta-analytically derived network model

**Figure 1:**
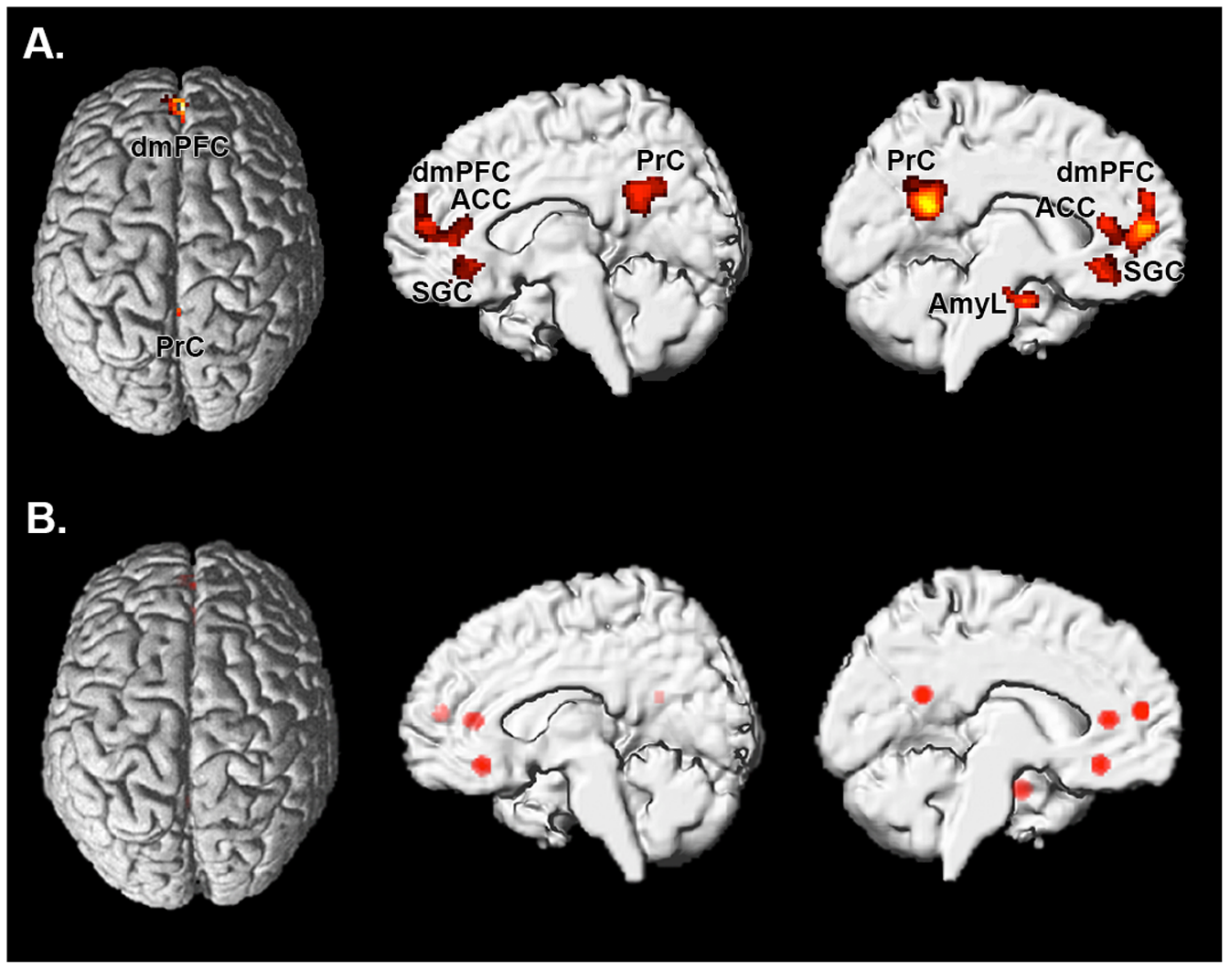
Significant results of the ALE meta-analysis delineating the ISA network (A; taken from^13^) and volumes of interest (VOIs) used for the functional connectivity analysis in healthy controls and patients (B). ACC: anterior cingulate cortex; AmyL: left Amygdala; dmPFC: dorsomedial prefrontal cortex; PrC: precuneus; SGC: subgenual cingulate cortex.

The ISA network model was based on two previous meta-analyses collectively involving more than 2,000 fMRI findings [13]. The meta-analytic approach used to define those brain regions that are reliably involved in affective and introspective tasks has been described in detail elsewhere [13]. In brief, we used the revised version of the activation likelihood estimation (ALE) approach for coordinate-based meta-analyses of neuroimaging results [17] to identify brain regions that are consistently implicated in affective and introspective processing, respectively, across a large number of experiments, resulting in a robust functional-anatomic model of the ISA network. In particular, we performed a conjunction analysis across two meta-analyses which investigated the statistical convergence of functional neuroimaging results for the so-called “default mode network” relevant for self-referential cognition or introspection (DMN) and emotional processing (EMO) across a large number of studies (DMN: n = 1474; EMO: n = 533) (for details see [13]). The resulting ISA network included the anterior cingulate cortex (ACC), left amygdala (AmyL), dorsomedial prefrontal cortex (dmPFC), precuneus (PrC) and subgenual cingulate cortex (SGC; see Figure 1A and Table 1). We furthermore performed a control analysis to ensure that the differences in connectivity between patients and controls were specific to affect processing and introspection and not part of a more general disease pattern. To this end, we used a network that has previously been associated with decoding speech [18] as speech perception should not be related to socio-affective aspects of depression. This network comprised left inferior frontal gyrus (IFG, area 44/45), the left middle temporal gyrus (MTG), the left angular gyrus (AG) and the left thalamus (Table 2).

**Table 1:** Activation peaks of ISA network (taken from^13^).

Macroanatomical location	MNI coordinates		
	x	y	z
Subgenual cingulate cortex (SGC)	0	32	−12
Anterior cingulate cortex (ACC)	0	36	10
Amygdala (AmyL)	−22	−6	−24
Precuneus (PrC)	−4	−54	22
Dorsomedial prefrontal cortex (dmPFC)	−2	52	14

**Table 2:** Activation peaks of control network (taken from^18^).

Macroanatomical location	MNI coordinates		
	x	y	z
Middle temporal gyrus (MTG)	−57	−27	−5
Inferior frontal gyrus (IFG)	−51	20	15
Thalamus	−6	−11	5
Angular gyrus (AG)	−48	−56	29

### Resting state fMRI data: imaging & preprocessing

Functional connectivity of the ISA and the language control network was investigated using resting state fMRI images acquired in 57 patients with depression and 57 age-, and gender-matched, healthy volunteers without any record of neurological or psychiatric disorders at two different sites (Aachen and Goettingen; for group characteristics see Table 3). Importantly, patients and controls were not only matched at the (overall) group level, but also within each site. That is, each of the two sites not only investigated an equal number of patients and controls, but within each site patients and controls were also carefully matched with respect to age and gender, thereby minimizing any potential bias. For all patients' diagnosis was confirmed by clinical examination of the attending psychiatrist in accordance with the International Classification of Diseases (ICD-10) and supplemented by the Beck Depression Inventory (BDI-II) as a self-report measurement of symptom severity [19]. Of the 57 examined patients, 27 were currently experiencing their first depressive episode (classified as F32 according to ICD-10), while the remaining 30 patients had had previous episodes and were, hence, classified as “recurrent depressive disorder” (F33 according to ICD-10). Participants were instructed to lie still during the scanning session and to let their mind wander, but not to fall asleep. The latter was confirmed during a post-scan debriefing interview.

**Table 3:** Group characteristics. *SD: standard deviation; M: male; F: female; BDI: Beck Depression Inventory II, HRSD: Hamilton Rating Scale for Depression*.

	Control group	Patient group
**Number of participants**		
Entire group	57	57
Aachen subgroup	30	30
Goettingen subgroup	27	27
**Gender ratio**	M: 30; F: 27	M: 30; F: 27
**Mean age** (SD)		
Entire group	36.74 (11.48)	36.89 (11.40)
Aachen subgroup	36.10 (12.58)	36.10 (12.21)
Goettingen subgroup	37.44 (10.56)	37.78 (10.82)
**Mean years of education** (SD)	13.78 (2.93)	13.81 (3.39)
**Mean BDI score** (SD)	1.33 (2.34)	20.12 (9.18)
**Mean HRSD score** (SD)	-	16.23 (10.07)
Aachen subgroup	-	12.20 (8.15)
Goettingen subgroup	-	20.7 (10.24)
**Mean age of depression onset** (SD)	-	25.74 (10.36)
**Mean depression duration in years** (SD)	-	9.09 (9.35)
**Mean number of depressive episodes** (SD)	-	3.23 (3.03)

It should be noted that all subjects received their regular medication as prescribed by the attending psychiatrist. While most patients were medicated with selective serotonin reuptake inhibitors (SSRI) or serotonin-norepinephrine reuptake inhibitors (SNRI), there was a considerable variability in the compounds used and many patients also received a combination therapy (see Table 4 for details). We were thus not able to perform sub-analyses between patients with different medication status, but rather regarded differences in medication and their potential effects on functional connectivity as a non-systematic source of variance in the patient group. In this context, it should be noted, that the variability in the patient pool potentially introduced by this heterogeneous medication should make it harder for the statistical analysis to identify consistent differences between patients and controls (due to the increase variance in the patient group). We would, thus, argue that our analysis represents a more conservative approach to identifying aberrant functional connectivity in patients with depression than a setting of homogeneous medication. In addition, this more natural setting should also allow better generalization to the overall population of patients with depression.

**Table 4:** Medication data for all patients.

Patient #	Medication - Antidepressants	Medication - Other
1	Venlafaxine	
2	Lithium	
3	Venlafaxine, Lithium	
4	Citalopram	
5	Duloxetine	
6	Citalopram	
7	Venlafaxine	
8	Duloxetine	Quetiapine
9	Opipramol, Sertraline, Mirtazapine	
10	Venlafaxine, Mirtazapine	
11	Reboxetine	
12	Sertraline	
13	Escitalprame	
14	Venlafaxine	
15	Amitriptylinoxid	Topiramate
16		Quetiapine
17	Venlafaxine	
18	Duloxetine	
19	Mirtazapine, Citalopram	
20	Mirtazapine	
21	Paroxetine	
22	Citaloprame	
23	Venlafaxine	
24	Bupropion	Quetiapine
25	Sertraline	
26	Mirtazapine, Venlafaxine	
27	Citalopram	
28	Mirtazapine	
29	Sertraline	
30	Venlafaxine, Mirtazapine	
31	Lithium, Escitalopram	Melperone
32	Venlafaxine, Mirtazapine	Pipamperone, Zopiclone
33	Escitalopram	Lorazepame
34	Escitalopram, Mirtazapine	
35	Duloxetine, Mirtazapine	
36	Venlafaxine, Mirtazapine	Quetiapine, Lorazepame
37		
38	Citalopram	
39	Escitaloprame, Agomelatine	Quetiapine, Zopiclone
40	Escitalopram	
41	Amitriptyline	Risperidone, Pregabaline
42	Escitalopram	Quetiapine
43	Fluoxetine	Quetiapine
44	Sertraline, Mirtazapine	
45		
46	Venlafaxine, Reboxetine	Valproic acid
47	Venlafaxine	
48		
49	Escitaloprame, Amitriptyline	
50	Citalopram	
51	Amitriptylin	Valproic acid
52	Venlafaxine, Mirtazapine	Olanzapine
53	Mirtazapine, Escitaloprame	
54		
55	Mirtazapine, Venlafaxine	
56	Mirtazapine	
57	Venlafaxine	

For each subject resting state EPI images were acquired using a standard blood-oxygen-level-dependent (BOLD) contrast [gradient-echo EPI pulse sequence] using highly similar sequences run on the same scanner type at both sites [Siemens “TIM Trio”]. In particular, the TE was 30 ms at both sides, while TR (Aachen: 2.2s, Goettingen 2s) and resolution (3 vs. 3.1 mm isotropic) were very similar. While there were more dynamics taken in Aachen (250) than Goettingen (156), these differences should not have a substantial impact on the estimation of functional connectivity in the current approach of estimating stationary connection parameters by time-series correlation. While it obviously would have been advantageous to have exactly matching protocols, this unfortunately was not possible in this retrospective pooling of data. While we acknowledge differences in acquisition as a weakness of our study, we would argue that this should not influence our results as we explicitly modeled and removed any potential site-effects prior to statistical inference. In this context, we would raise attention again to the fact, that patients and controls were closely matched within each site. This balanced design, therefore, allows estimating and removing any global site-effect that may relate to scanner and parameters. Moreover, we closely examined the site-wise connectivity parameters of patients and controls for all connections that became significant in the statistical analysis. In summary, while our retrospective pooling approach did not allow us to perfectly match acquisition parameters, the well-balanced setup from the close matching within each site allowed excluding any influence of these differences on the results.

Prior to further processing (using SPM8, www.fil.ion.ucl.ac.uk/spm) the first four images were discarded allowing for magnetic field saturation. The EPI images were first corrected for head movement by affine registration using a two-pass procedure. The mean EPI image for each subject was then spatially normalized to the MNI single subject template using the unified segmentation approach, the ensuing deformation field was applied to the individual EPI volumes and the output images were smoothed by a 5-mm FWHM Gaussian kernel.

**Figure 2:**
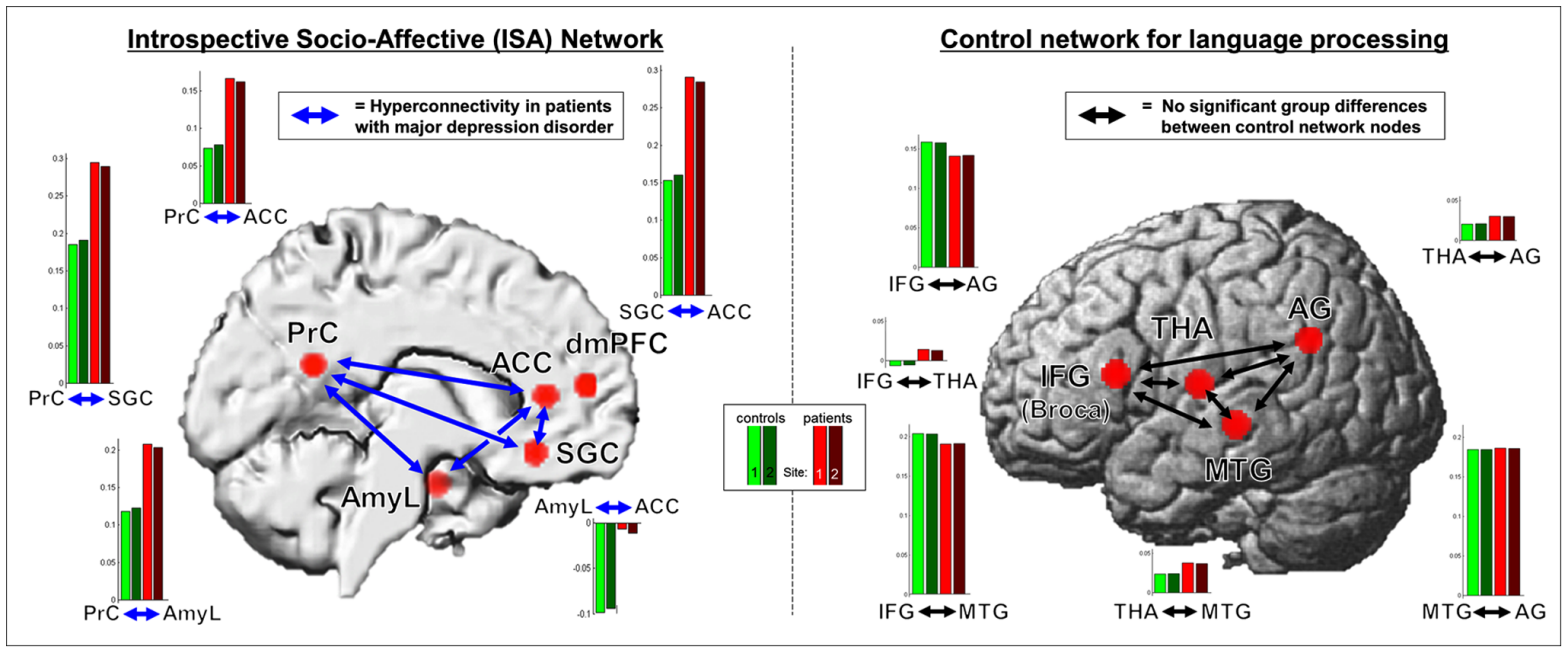
Results of the resting state functional connectivity analysis in the control and patient group for ISA (left) and control network (right). Bar plots depict measures of functional connectivity for patients (red) and controls (green) across the two different measurement sites (light colors: Aachen; dark colors: Goettingen).

In order to reduce spurious correlations by confounds such as physiological noise and motion, variance that could be explained by first- or second-order effects of the following nuisance variables was removed from each voxel's time series: i) the six motion parameters derived from the image realignment ii) their first derivative iii) mean grey, white matter and CSF signal intensity. These corrections have been shown to increase specificity and sensitivity of the analyses and may be used to robustly identify group-differences in resting-state functional connectivity [20]–[22]. Furthermore, we also compared movement parameters across the two diagnostic groups and were, thus, able to demonstrate that no group-specific differences in head motion exist in our data set (Table 5). Data was then band pass filtered preserving frequencies between 0.01 and 0.08 Hz [22]. The time course for each of the brain regions identified in the meta-analysis described above (Figure 2, Table 1) or in the control analysis (language network; Figure 2, Table 2) was extracted for each subject as the first eigenvariate of all grey-matter voxels located within 5 mm of the respective peak coordinate (Figure 2, Tables 1 & 2).

**Table 5:** Comparison of motion parameters across groups and sites. *SD: standard deviation, DVARS: temporal derivative of timecourses (cf. Power et al. 2012), FD: framewise displacement (cf. van Dijck et al. 2012), RMS: variance over voxels (cf. Satterthwaite et al. 2013).*

	Control group	Patient group	Statistical comparison (p-values) *t-test/ranksum*
**DVARS**			
Entire group mean (SD)	1.37 (0.28)	1.42 (0.32)	0.4346/0.4771
Aachen subgroup mean (SD)	1.19 (0.17)	1.30 (0.29)	0.1007/0.2394
Goettingen subgroup mean (SD)	1.55 (0.26)	1.52 (0.32)	0.7022/0.6836
**FD**			
Entire group mean (SD)	0.28 (0.12)	0.30 (0.18)	0.4427/0.7256
Aachen subgroup mean (SD)	0.25 (0.12)	0.32 (0.23)	0.1617/0.1945
Goettingen subgroup mean (SD)	0.31 (0.12)	0.28 (0.14)	0.5233/0.5175
**RMS**			
Entire group mean (SD)	0.20 (0.09)	0.22 (0.14)	0.3499/0.6861
Aachen subgroup mean (SD)	0.18 (0.08)	0.24 (0.18)	0.1454/0.1772
Goettingen subgroup mean (SD)	0.22 (0.08)	0.21 (0.10)	0.5961/0.4869

### Resting state fMRI data: individual & group level analyses

For each subject and analysis, ISA or language network, we computed linear (Pearson) correlation coefficients between extracted time series. These voxel-wise correlation coefficients were then transformed into Fisher's z scores representing the functional connectivity for each connection in each subject. As part of a confound-removal procedure, any variance that could be explained by the factor ‘site’ (Aachen/Goettingen) and its interaction with diagnosis was removed from these scores. Hereby we accommodated for potential differences between sites caused, e.g. by scanner setup, in spite of identical hardware and similar protocols. This approach, thus, capitalizes on the balanced setup and close matching between patients and controls within each site and allowed to focus the subsequent statistical analysis on the main effect of diagnosis. In other words, in the present analysis, given the balanced design we were able to first remove all potentially confounding effects in the distribution of connectivity strength before focusing the inference aberrant connectivity in patients across sites. Group comparison between patients and controls was then performed by a non-parametric approach using 10,000 realizations of the null hypothesis (group label exchangeability) in a Monte-Carlo simulation [23], [24]. Results were regarded as significant if they exceeded a posterior probability of P>0.95 (equivalent to p<0.05) that the 'real' group difference is bigger than a simulated random difference, following FDR-correction for multiple comparisons.

### Relations to clinical characteristics

In order to assess the possible effects of disease onset and duration we performed additional analyses, which compared functional connectivity in subgroups of our patient sample using the same statistical approach as used for the main analysis between patients and controls. The subgroups were assembled by using a median split procedure which differentiates between those patients with early and late depression onset and, secondly, with short and long disease duration. To assess a possible relationship between functional connectivity and symptom severity as indexed by BDI scores Spearman rank-correlation analyses were used.

## Results

### Functional connectivity differences in ISA network

Resting-state functional connectivity differences between patients with depression and healthy controls were assessed between all nodes of the introspective socio-affective (ISA; Figure 1) network, namely between anterior cingulate cortex (ACC), left amygdala (AmyL), dorsomedial prefrontal cortex (dmPFC), precuneus (PrC) and subgenual cingulate cortex (SGC). Significant differences (p<0.05, FDR corrected for multiple comparisons across all connections) in resting state functional connectivity (RSFC) between patients and controls were found along several edges of this network, with patients showing higher neurofunctional coupling than control participants in all cases. In particular, patients with depression demonstrated increased functional connectivity (more positive) compared to controls between ACC and the PrC (p<0.001, Cohen's d: 0.41), AmyL and PrC (p<0.001, Cohen's d: 0.46), SGC and ACC (p<0.001, Cohen's d: 0.44) and between SGC and PrC (p<0.001, Cohen's d: 0.47) (see Figure 2 & Supplementary Figures S1-S10 in File S1). Furthermore, higher (less negative) functional connectivity was also observed in patients between ACC and the AmyL than in the control group (p<0.004, Cohen's d: 0.49). Group differences between the remaining other connections did not reach statistical significance. There were no connections showing decreased functional connectivity in the patients, even when performing inference at an uncorrected level. All of the observed effects were extremely similar across sites (see bar plots in Figure 2 & Supplementary Figures in File S1).

In order to further qualify the findings described above, within-group effects of functional connectivity were also calculated. These calculations demonstrate that hyperconnectivity in the group comparison results from a functional connectivity increase in the patient as compared to the control group along connections that already showed a significant (p<0.05, FDR corrected) coupling in the latter group, with the exception of the connection between ACC and AmyL (which was significantly negative in controls). That is, for all connections apart from ACC <–> AmyL a significant (positive) coupling in healthy controls was furthermore significantly increased in the patients. These observed effects thus reflect a true hyper-connectivity of the respective connections in the patients with depression. For the last connection (ACC <–> AmyL), the control group showed a significant negative coupling, while there was no such coupling in the patient group (Figure 2 & Supplementary Figures in File S1). That is, the significant anti-correlation in controls was no longer observed in patients for this connection.

### Functional connectivity differences in control network

For the language-related control network, no differences in connectivity between patients and controls were observed (see Figure 2), even when repeating the analysis without correction for multiple comparisons. This negative finding makes it highly unlikely that the experimental findings above may stem from generalized disturbances of functional connectivity or systematic confounds.

### Relation to clinical characteristics

**Figure 3:**
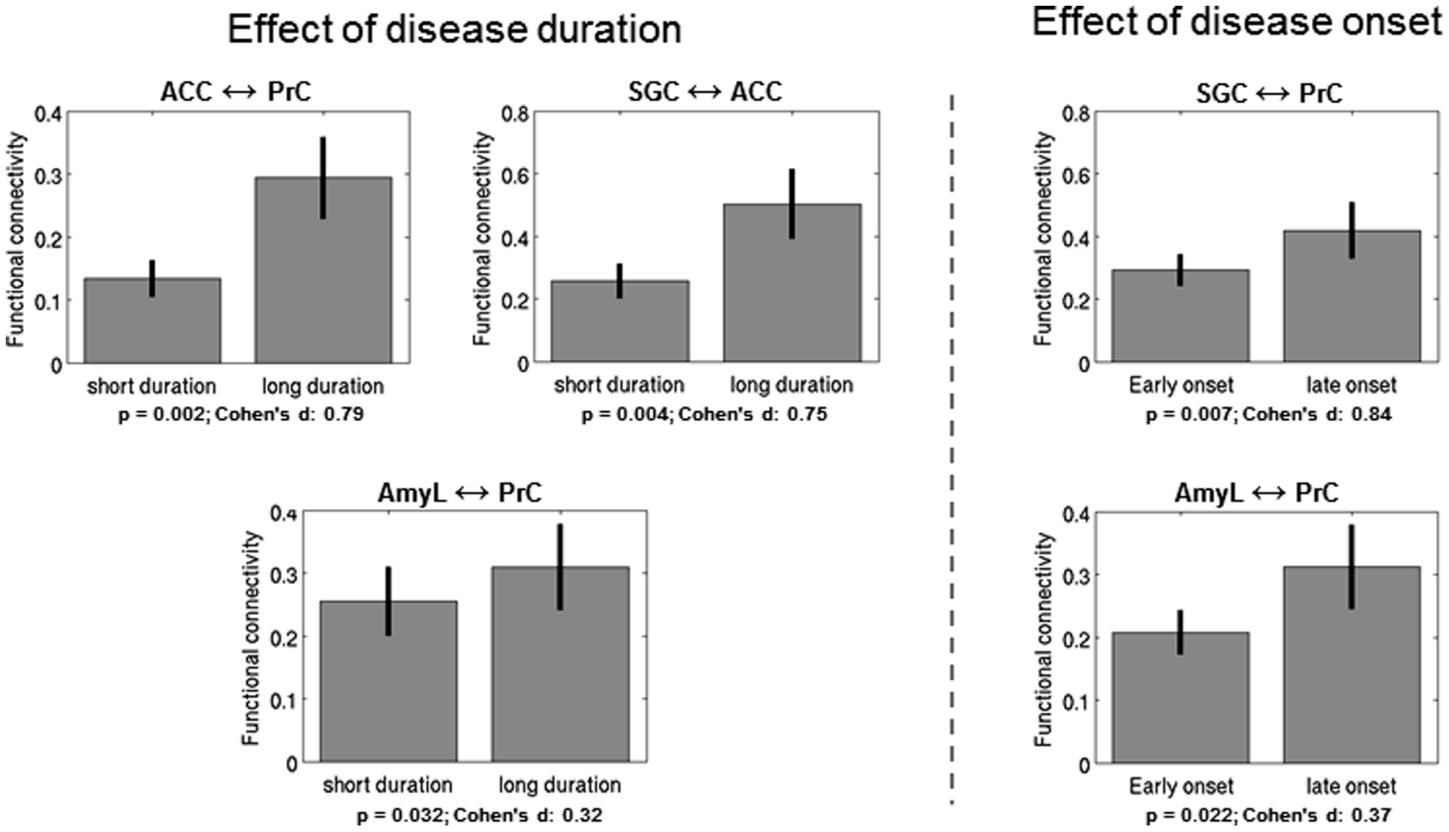
Significant results of the comparison of functional connectivity between patient subgroups of short and long disease duration and early and late onset of illness. Subgroups were defined each by a median split of the patient group with ≥5 years for long duration and ≥24 years of age for late disease onset. ACC: anterior cingulate cortex; AmyL: left amygdala; PrC: precuneus; SGC: subgenual cingulate cortex.

When comparing patient subgroups of short and long duration (≥5 years) of illness (see Figure 3), functional connectivity between ACC and PrC and between ACC and SGC was significantly higher in the subgroup with longer disease history. Patients with later onset (age≥24) of depression showed higher connectivity between SGC and PrC in comparison to the group with early onset. Finally, the functional coupling between PrC and AmyL (which was significantly negative in controls) was increased (i.e. became non-significantly different from zero) for both groups with long duration and late onset of disease.

Spearman rank-correlation analyses conducted to assess a possible relationship between functional connectivity and symptom severity did not yield any statistically significant results.

## Discussion

Here, we used a model-based approach to investigate resting state functional connectivity in a robust, meta-analytically defined introspective socio-affective (ISA) network in patients with depression and healthy controls. This investigation was based on the idea that depression is characterized by affective symptoms and alterations of self-referential cognition and introspection, which together aversely affect social interaction [4], [6]. Our analysis approach allows circumventing problems with the identification of relevant neural networks for the purpose of functional connectivity analyses [10] and provides an unbiased, yet functionally specific investigation of the ISA network in depression. Our analysis demonstrates striking, region-specific differences in functional connectivity profiles of the ISA network when comparing the control and the patient group. As a key finding, our analysis demonstrates markedly higher connectivity in individuals with depression as compared to healthy individuals, which is consistent with an emerging “hyperconnectivity hypothesis” of depression [16] (Figure 2). Importantly, absence of significant group effects in the control analysis demonstrates that the observed differences are specific to the introspection and socio-affective network and not manifestations of a more general pathology. Furthermore, the control analysis also indicates that preprocessing most likely did not introduce any artificial group differences. Finally, the absence of group differences for the control network also makes it unlikely that antidepressant medication of the patient group may have produced non-specific group differences. Combining the significant differences between patients and controls while controlling for multiple comparisons, the observation that significant coupling was observed for all but one affected connection in healthy controls and the fact that there was no difference in the control network even at an uncorrected level of statistical analysis strongly suggests a specific hyper-connectivity of a neural network relevant for social interaction in patients with depression.

One of the connections within the ISA network that is significantly increased in patients is the coupling between ACC and the PrC. This is consistent with previous research demonstrating elevated resting state connectivity within the so-called “default mode network” in depression [8], a network that has been related to self-referential cognition, introspection and prospective cognition [12], [13]. Hyperconnectivity of this network may specifically contribute to alterations of introspection and rumination in depression. Following the idea by Schacter et al. [25] that ‘prospective memory’, i.e. the imagination and projection of future events, may be mediated by these regions, the observed aberrations may also plausibly relate to the loss of perspective, another core symptom of depression closely related to rumination. As ‘prospective memory’ has been related to the posterior midline, dysconnectivity of PrC may, thus, underlie the difficulties with optimistic future-oriented thoughts and the ruminations typical of affective disorders. An additional analysis targeting the effect of disease duration demonstrated that long as compared to short duration leads to a more pronounced increase in functional connectivity of this region, which is consistent with the notion that chronic forms of depression might be most strongly linked to feelings of helplessness extending to future thoughts and social interactions [4].

In patients, the PrC also showed increased coupling with the amygdala, a result that resembles previous findings of the effects of social stress on functional connectivity [26]. Here, increased connectivity between amygdala, posterior cingulate cortex and precuneus in response to social stress has been related to the “processing and regulation of emotions”. Connectivity changes between these regions might, therefore, constitute a neural marker for vulnerability to stress [26]. On the other hand, the precuneus has also been linked to autobiographical memory, self-reflection and mentalization, processes which are known to contribute to emotion regulation [27].

Furthermore, our analysis demonstrated hyperconnectivity between SGC and ACC as well as SGC and the PrC. The SGC, in particular, has been implicated as a neurofunctional ‘hot spot’ of affective disorders. Seminal studies by Mayberg and colleagues have demonstrated evidence for hyperactivity of SGC in treatment-resistant populations of depressed patients [28] and have documented that deep brain stimulation (DBS) of subgenual cingulate white matter results in remission in some previously treatment-resistant patients [29]. Consistently, recent evidence demonstrates that electroconvulsive therapy (ECT) in major depression weakens symptomatology while reducing serotonin-1A receptor binding in the subgenual part of cingulate cortex [30] as well as decreasing left dorsolateral prefrontal connectivity [16]. Transcranial magnetic stimulation over dorsolateral prefrontal cortex (DLPFC) also appears to be effective by indirectly influencing SGC, whose activity is anti-correlated to DLPFC [31]. Furthermore, differences in the serotonin transporter genotype (5-HTTLPR) have been shown to modulate brain responses to emotional faces such that non-depressed, short variant homozygotes demonstrate an absence of subgenual deactivation, which parallels findings in clinically depressed individuals [32]. Activation differences in SGC in response to social stimuli are also linked to differences in empathic concern [33], a failure of which may perpetuate social interaction difficulties. The results of the current study, therefore, are in line with, but also extend previous findings by showing that depression is not only associated with SGC hyperactivity but also with hyperconnectivity of SGC to ACC and PrC.

Finally, our analysis also demonstrated significant connectivity differences between patients and controls in the connection of ACC and AmyL: while controls exhibit a significant anti-correlation between the two brain regions, no such pattern is observed in the patient group. That is, in contrast to the aforementioned connections, which increased their (in controls positive) coupling, significantly increased connectivity along the ACC <–> AmyL connection reflects a loss of anti-correlation in patients. Importantly, functional coupling of these two regions is known to play a crucial role in the experience and modulation of affect and alterations thereof may, hence, relate to negative affect and deficits in emotion regulation as key factors in affective disorders [34], [35]. In line with this view, personality traits related to affective disorders have been shown to correlate with aberrant ACC-amygdala connectivity [35], [36]. Our finding of hyperconnectivity between these regions is moreover consistent with recent MEG evidence for increased ACC-amygdala connectivity in major depression [37]. Conversely, antidepressant drugs, which affect the serotonergic or noradrenergic system, have been shown to reduce resting state connectivity between the amygdala and frontal regions as compared to placebo [38], which might help to restore the anti-correlations seen in controls. Also, it has been shown that genetic polymorphisms, which impact cerebral serotonin turnover, influence cingulate-amygdala interactions [34]. The suggestion of a functional optimum of interregional connectivity may also help to explain why a connectivity increase in the case of pathology -as observed in our patient sample- may actually lead to a decrease in function.

The use of a meta-analytic approach to identify neural networks thus allows the selection of the relevant brain regions to be based on *a priori* hypotheses and statistically converging neuroimaging evidence. The brain regions selected may, however, still be involved in other, possibly unrelated psychological processes. Recent advances in neuroimaging databases have attempted to address this issue by allowing to perform functional decoding, i.e. providing a quantification of the probability of a particular psychological function given a certain activation pattern. We have performed functional decoding for the different regions of the ISA network using the BrainMap database (brainmap.org). As expected, the results of this additional analysis highlight the involvement of the different regions in emotion- and introspection-/social cognition-related processes (see Supplementary Figures in File S1).

In terms of an outlook to future research, we would suggest that using model-based analyses of resting state fMRI data and applying *a priori* defined meta-analytically derived regions of interest could be a clinically feasible, standardized and, therefore, powerful way to document treatment-induced changes at the neural level. This standardized approach might help to shed new light on the effectiveness and selection criteria of psychotherapeutic, drug-based and electroconvulsive treatments and could be developed towards the prediction of treatment outcome. Future studies should also include resting state scans of patients before treatment and during treatment in order to further explore the relationship between clinical symptoms and functional connectivity changes in the ISA network over the course of the illness. Obviously, our cross-sectional study can only provide limited information in this respect. Furthermore, using a multi-modal approach that includes both neurofunctional and neuroanatomical measures would allow to also take into consideration putative grey matter differences between diagnostic groups, which could affect the relationship between clinical symptomatology and neurofunctional coupling. Here, the use of volumetric measures could inform partial correlation analyses to investigate the degree of association between functional connectivity and symptom severity, with the effect of grey matter differences removed.

## Supporting Information

File S1 **Figures S1–S14.**
(DOC)
