# Supplementary material for: Meta-Analytically Informed Network Analysis of Resting State fMRI Reveals Hyperconnectivity in an Introspective Socio-Affective Network in Depression
Source: PLoS One. 2014 Apr 23;9(4):e94973. doi: 10.1371/journal.pone.0094973 (PMC3997658; doi:10.1371/journal.pone.0094973)
Supplement: File S1 — Figures S1–S14. (DOC) [file pone.0094973.s001.doc]

**Supplementary Figures**

*
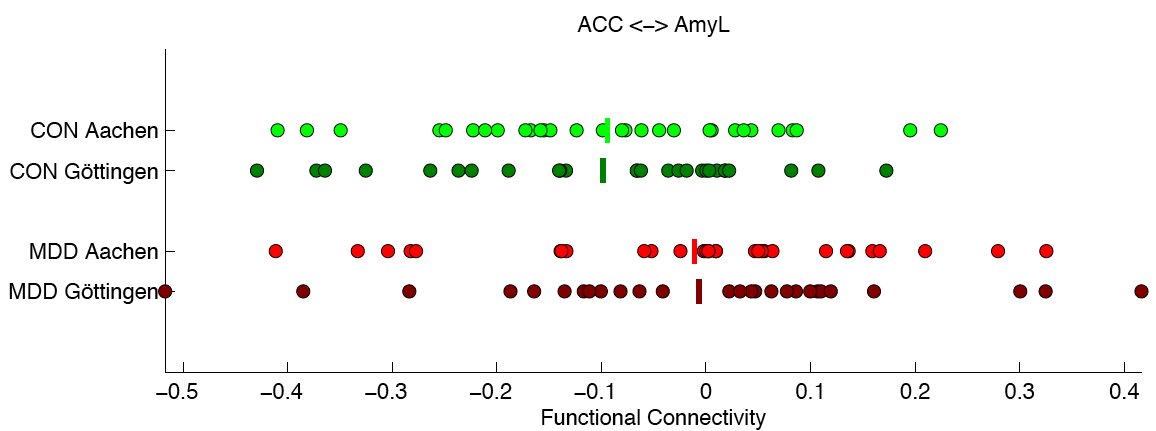
*

**Figure S1:** Scatterplot of functional connectivity across groups and sites for the connection of anterior cingulate cortex and amygdala.

*
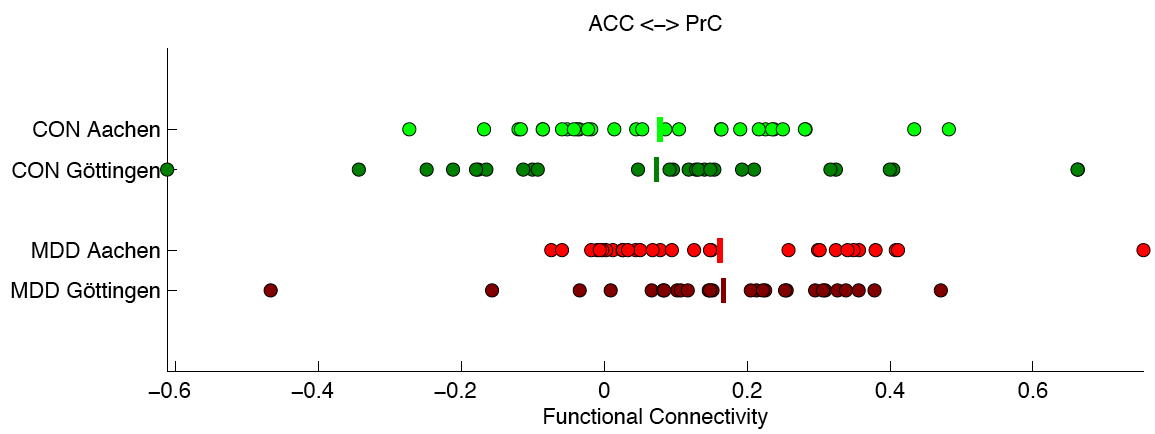
*

**Figure S2:** Scatterplot of functional connectivity across groups and sites for the connection of anterior cingulate cortex and precuneus.

*
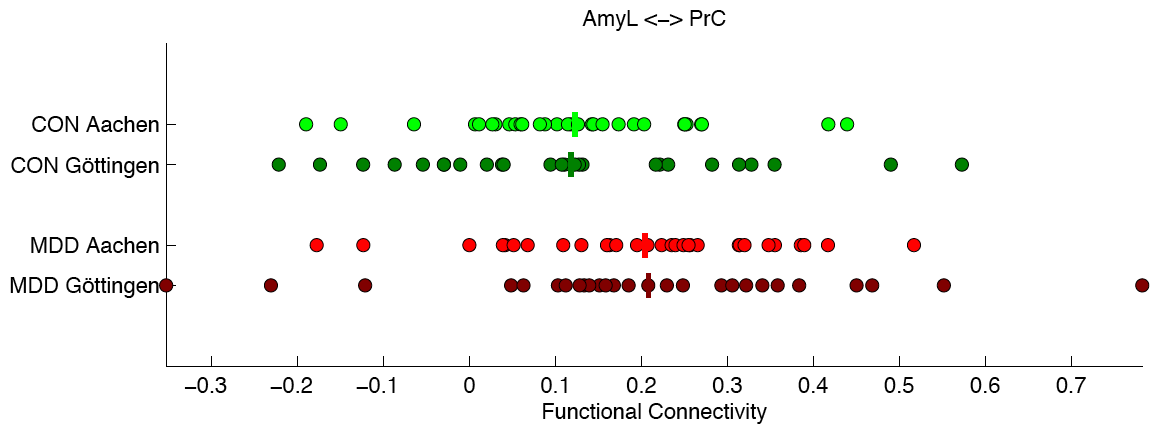
*

**Figure S3:** Scatterplot of functional connectivity across groups and sites for the connection of amygdala and precuneus.

*
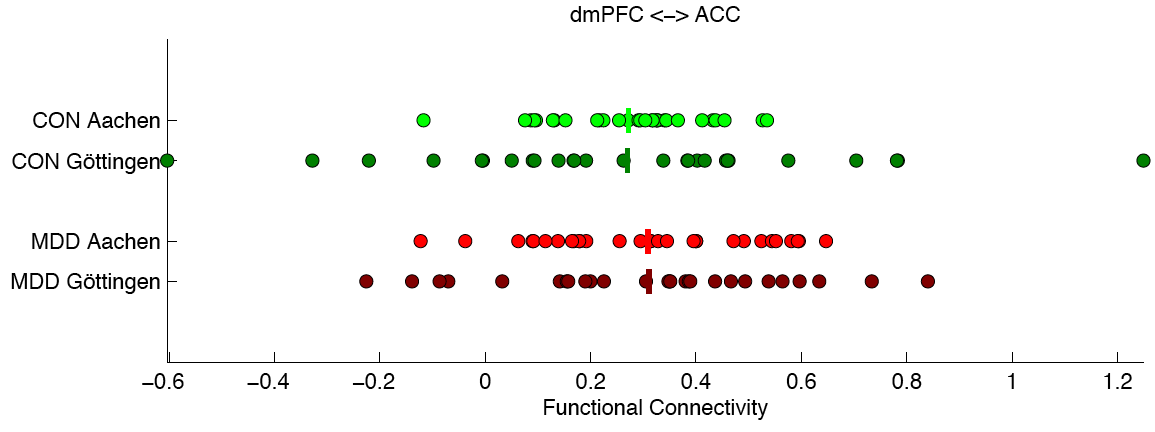
*

**Figure S4:** Scatterplot of functional connectivity across groups and sites for the connection of dorso-medial prefrontal cortex and anterior cingulate cortex.

*
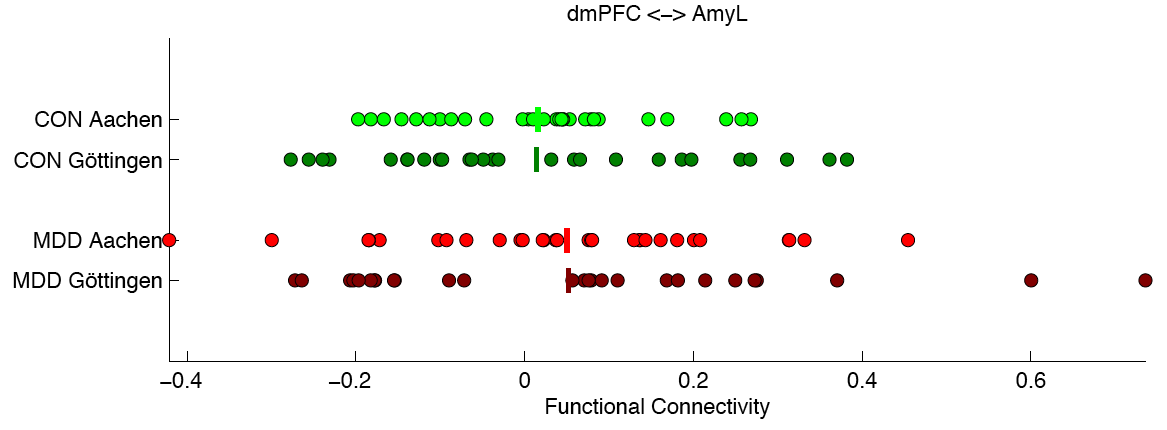
*

**Figure S5:** Scatterplot of functional connectivity across groups and sites for the connection of dorso-medial prefrontal cortex and amygdala.

*
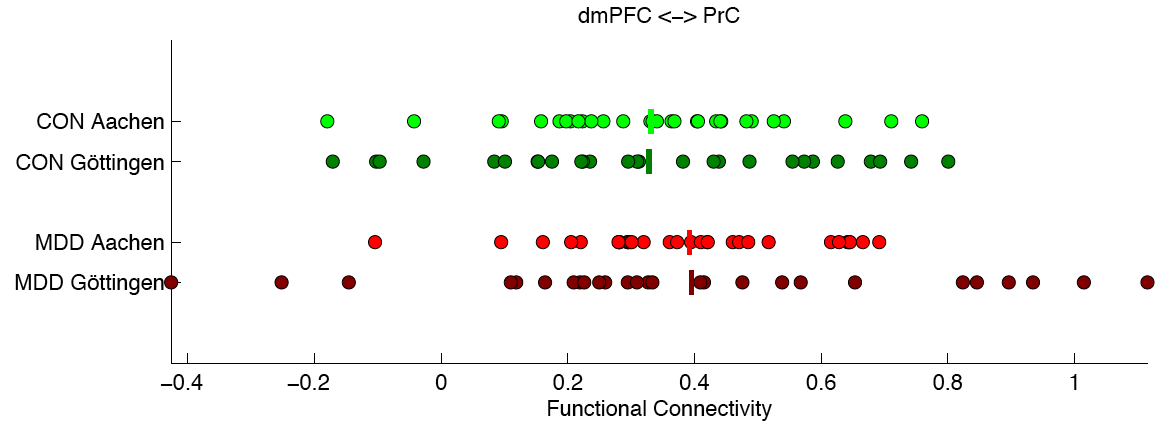
*

**Figure S6:** Scatterplot of functional connectivity across groups and sites for the connection of dorso-medial prefrontal cortex and precuneus.

*
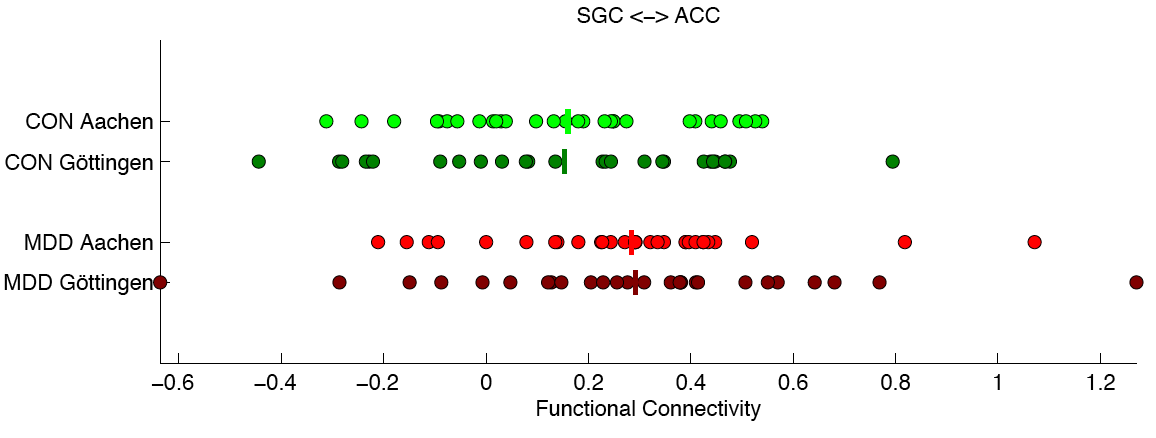
*

**Figure S7:** Scatterplot of functional connectivity across groups and sites for the connection of subgenual cingulate cortex and anterior cingulate cortex.

*
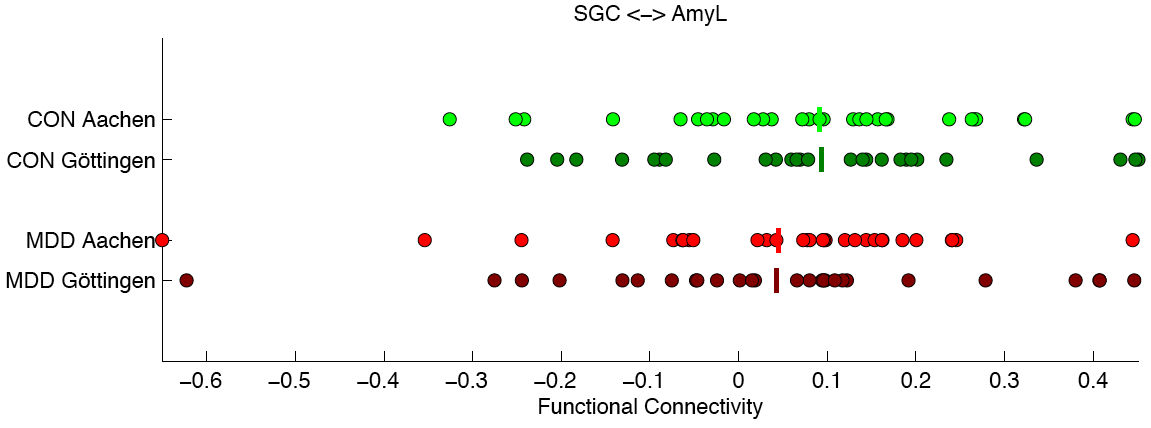
*

**Figure S8:** Scatterplot of functional connectivity across groups and sites for the connection of subgenual cingulate cortex and anterior cingulate cortex.

*
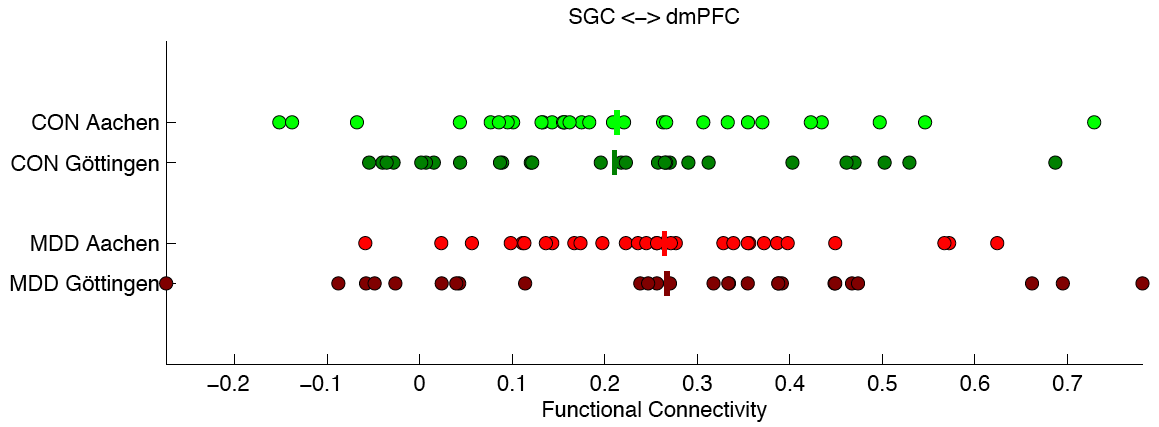
*

**Figure S9:** Scatterplot of functional connectivity across groups and sites for the connection of subgenual cingulate cortex and dorso-medial prefrontal cortex.

*
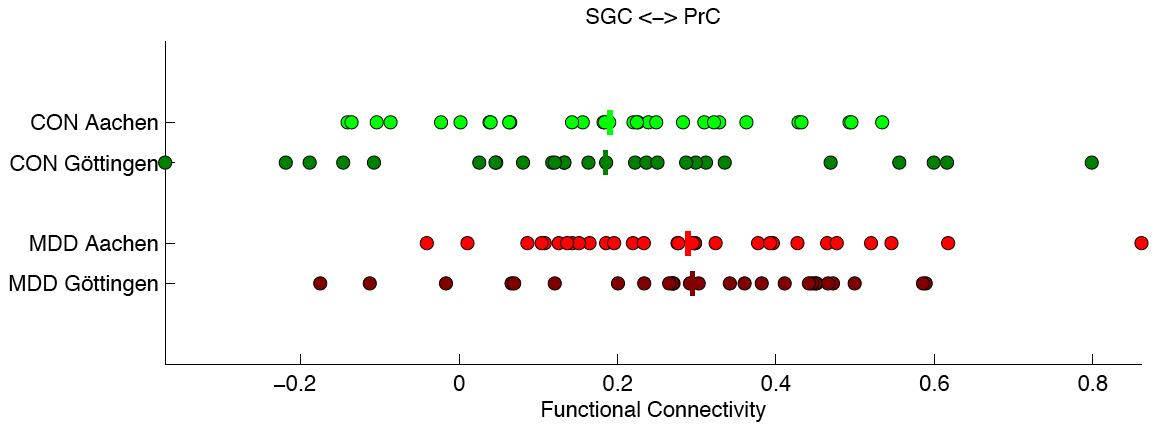
*

**Figure S10:** Scatterplot of functional connectivity across groups and sites for the connection of subgenual cingulate cortex and precuneus.


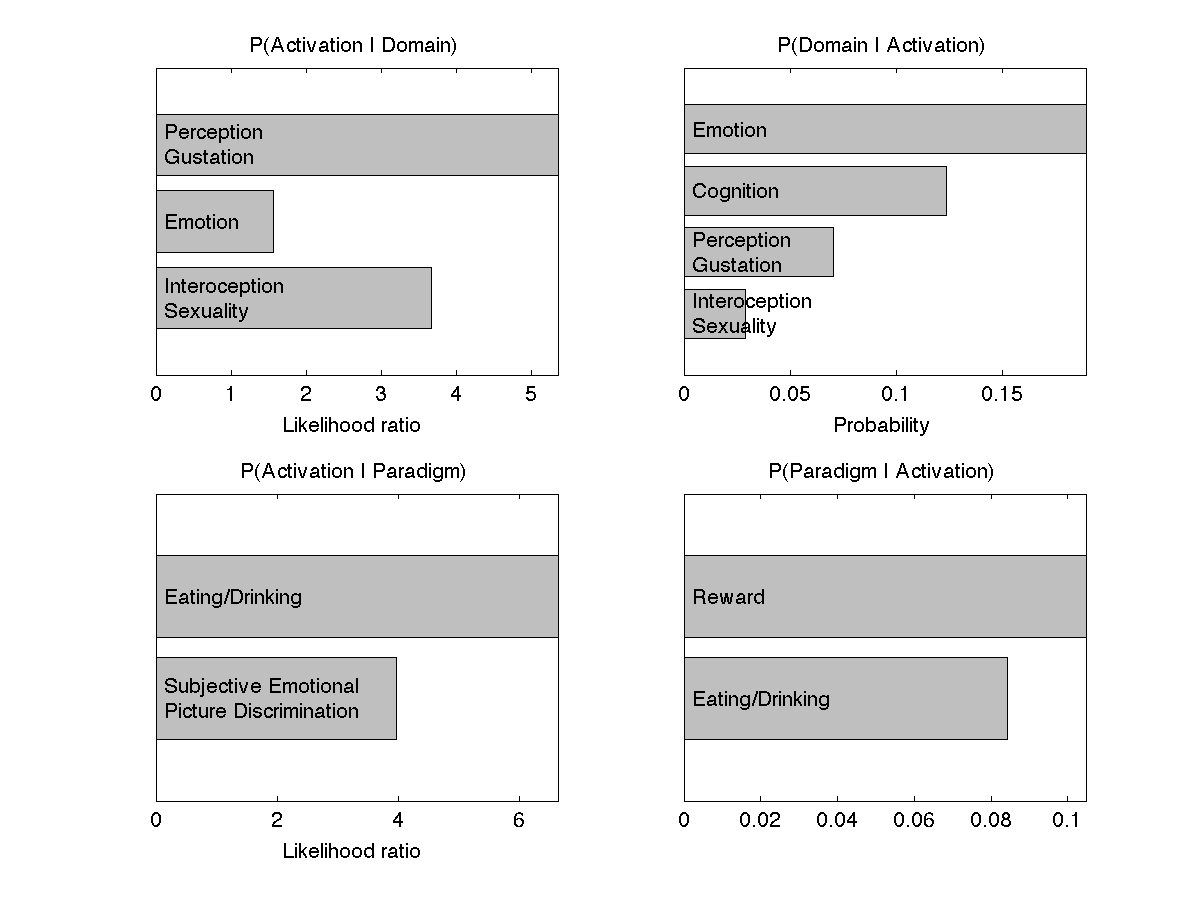


**Figure S11.** Functional decoding of the anterior cingulate cortex (ACC) using the BrainMap database.


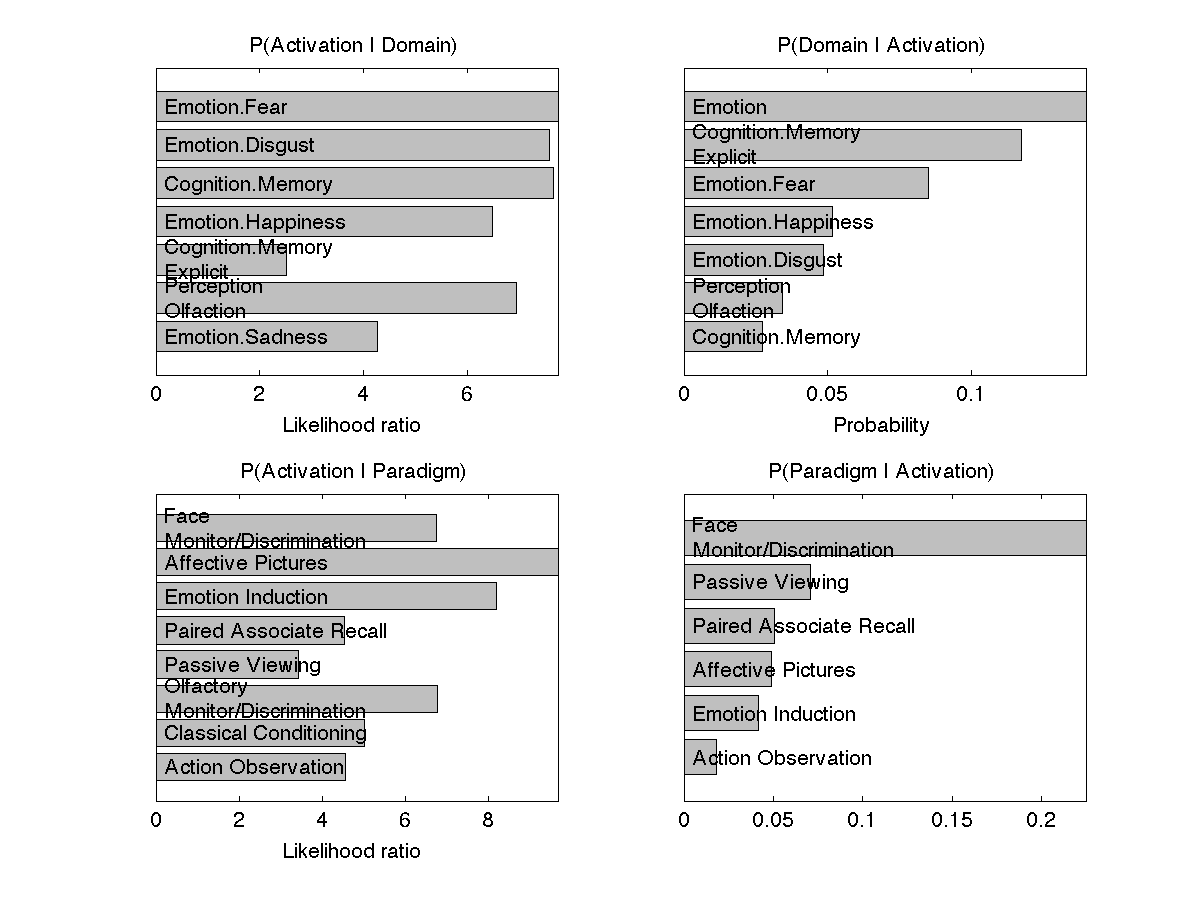


**Figure S12.** Functional decoding of the amygdala (Amy) using the BrainMap database.


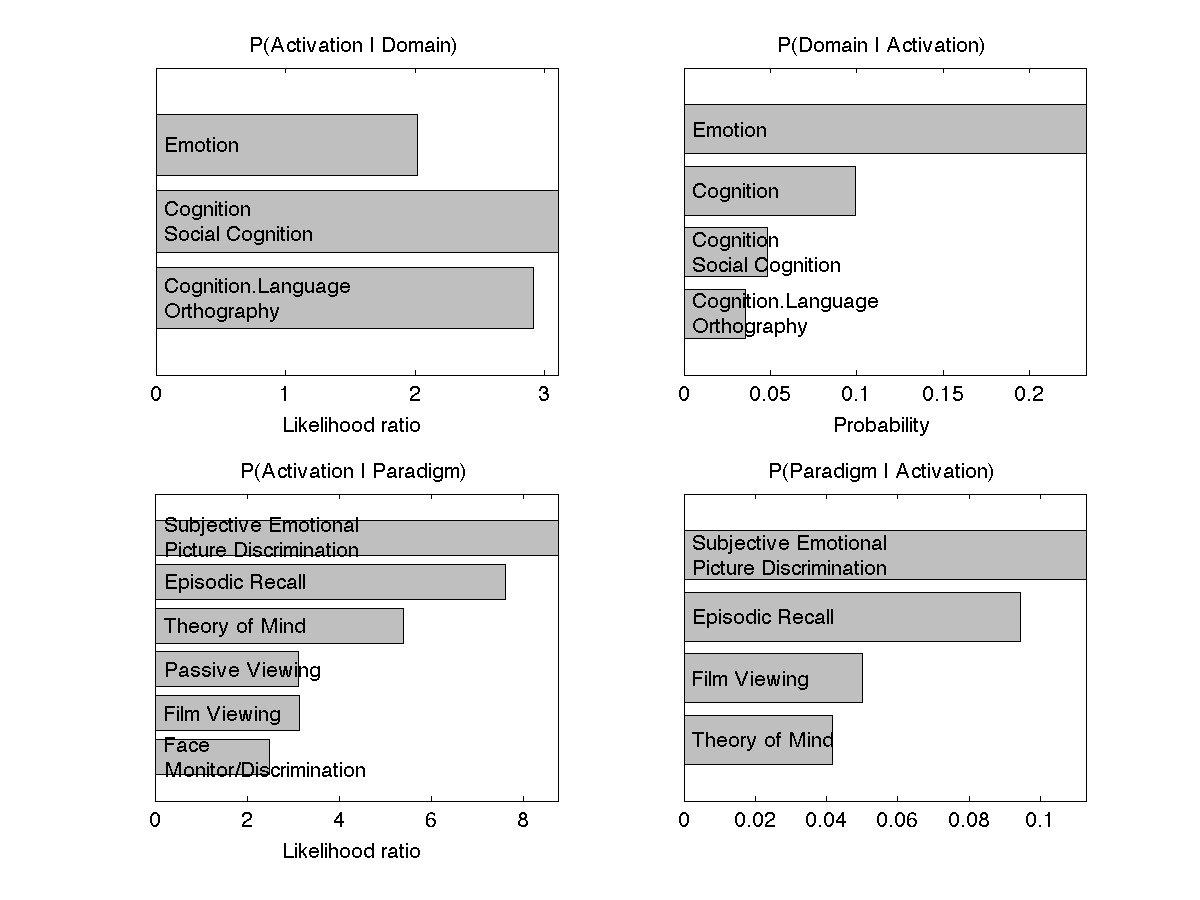


**Figure S13.** Functional decoding of dorso-medial prefrontal cortex (dMPFC) using the BrainMap database.


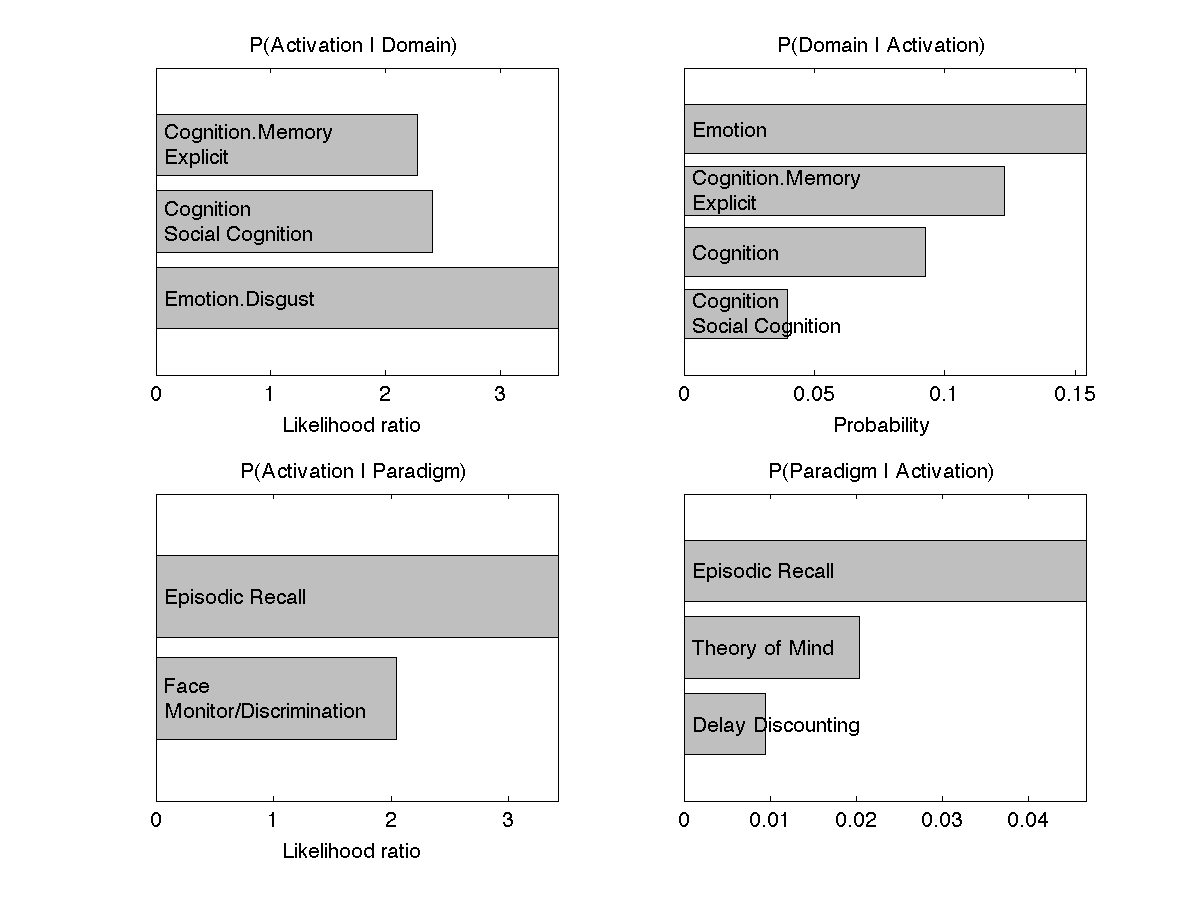


**Figure S14.** Functional decoding of the precuneus (PrC) using the BrainMap database.


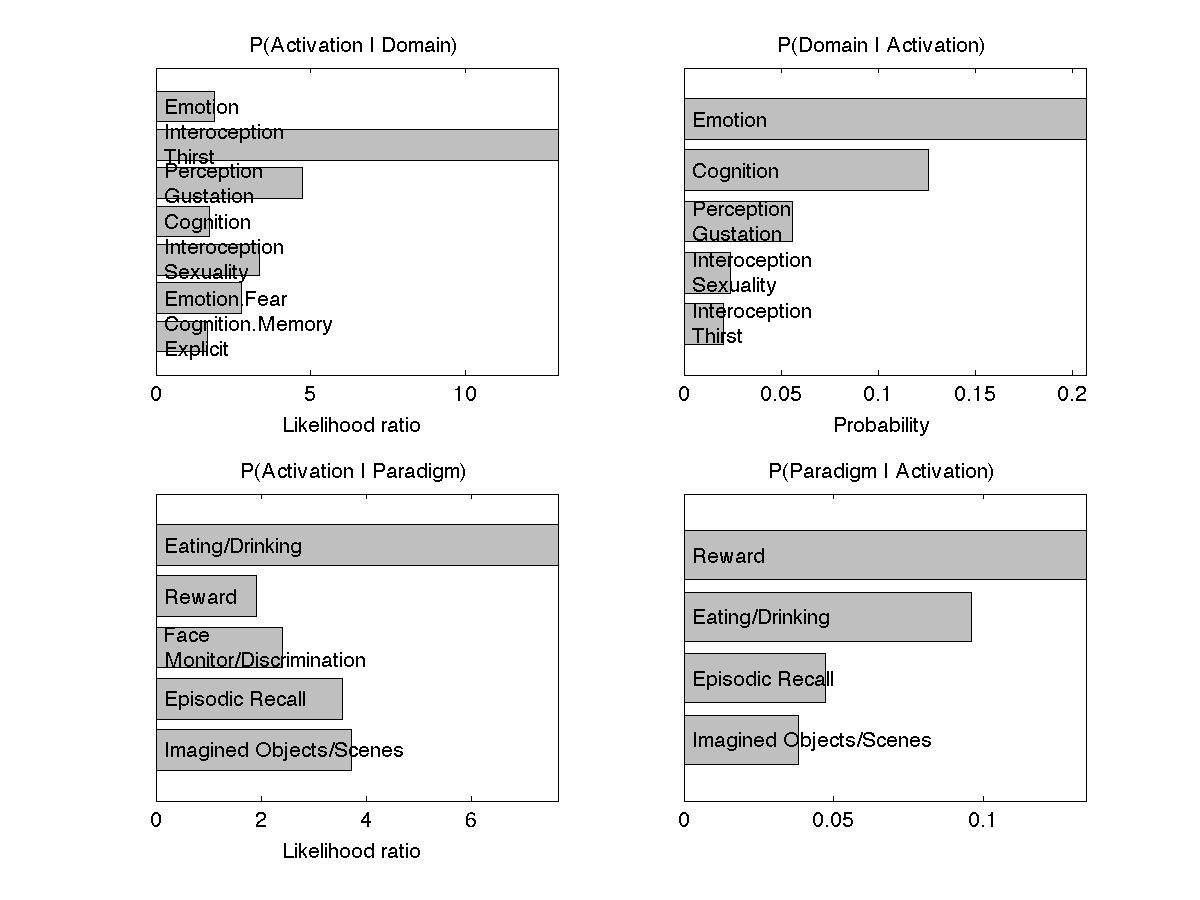


**Figure S15.** Functional decoding of subgenual cingulate cortex (SGC) using the BrainMap database.
